# Development of a large animal orthotopic intestinal transplantation model with long-term survival for study of immunologic outcomes

**DOI:** 10.3389/frtra.2024.1367486

**Published:** 2024-05-02

**Authors:** Sarah Merl, Bryan Chen, M. Esad Gunes, Hussein Atta, Kryscilla Yang, Dilrukshi Ekanayake-Alper, Dominik Hajosi, Fei Huang, Brittany Bhola, Satyajit Patwardhan, Philip Jordache, Greg Nowak, Mercedes Martinez, Tomoaki Kato, Megan Sykes, Kazuhiko Yamada, Joshua Weiner

**Affiliations:** ^1^ Columbia Center for Translational Immunology, Columbia University, New York, NY, United States; ^2^ Department of Pathology and Cell Biology, Columbia University, New York, NY, United States; ^3^ Department of Comparative Medicine, Yale School of Medicine, Yale University, New Haven, CT, United States; ^4^ Department of Surgery, Karolinska Institute, Solna, Sweden; ^5^ Department of Pediatrics, New York-Presbyterian Hospital, Columbia University, New York, NY, United States; ^6^ Department of Surgery, New York-Presbyterian Hospital, Columbia University, New York, NY, United States; ^7^ Department of Medicine, New York-Presbyterian Hospital, Columbia University, New York, NY, United States; ^8^ Department of Microbiology & Immunology, Columbia University, New York, NY, United States; ^9^ Department of Surgery, Johns Hopkins University, Baltimore, MD, United States

**Keywords:** swine, intestine, transplant, model, orthotopic

## Abstract

**Introduction:**

Intestinal transplantation (ITx) is the last remaining therapy for patients with intestinal failure once parenteral nutrition is no longer an option, however its use is limited by immunological complications, including high rates of rejection and morbidity associated with immunosuppression, such as infection and malignancy. We aimed to develop a large animal model of ITx with which to study the immune response to ITx and to design and test tolerance induction regimens.

**Methods:**

Learning from prior complications, we developed and progressively improved both surgical methods for the donor and recipient as well as postoperative management strategies. Methods of stoma generation, bowel positioning, vessel preparation, and fluid management were optimized. The immunosuppression strategy mirrored our clinical regimen.

**Results:**

As a result of our modifications, results improved from survival less than 1 month to consistent long-term survival with good graft function. We review several techniques that were developed to avoid pitfalls that were encountered, which can be used to optimize outcomes in this model.

**Discussion:**

Achieving long-term survival after swine orthotopic ITx permits immunological analysis and pre-clinical trials in a large animal model of ITx.

## Introduction

Intestinal transplantation (ITx) is a well-accepted therapy for individuals suffering from irreversible intestinal failure (IF), a condition characterized by the inability to maintain adequate nutrition and hydration through the digestive system due to inadequate length or impaired function of the bowel (1). However, intestinal grafts have the highest rate of rejection among solid organ transplants, which necessitates higher levels of immunosuppression. This causes increased risk of complications, such as infection, graft-vs.-host disease, and malignancy (2). As a result, fewer than 100 ITx are performed per year in the United States (3). Therefore, improving outcomes could make ITx a more attractive option for patients with IF.

With so few transplants performed, and with the limitations of experimentation on human subjects, a swine model provides an ideal platform to study the pathophysiology of ITx rejection. Swine are advantageous due to their large litter size, short time to maturity, anatomical and physiological similarity to humans, decreased ethical concerns relative to primate research, and well-established immunological models (4). Previously, intestine transplants have been performed in swine to investigate technical aspects of the surgery, pharmacology of immunosuppressive agents, characterization of rejection, presentation of ischemia reperfusion injury, nutritional aspects, biomarker monitoring, and concurrent bone marrow transplantation, but almost all have been short-term studies (less than one month). A very small number of studies with long-term survival have been carried out using heterotopically placed grafts (5) and orthotopic grafts (6–10), but these references do not provide significant detail about the surgical techniques and postoperative medical management, making the models difficult to reproduce.

We herein report our methods and important modification we made to achieve routine long-term post-transplant survival in a porcine model of orthotopic intestinal transplantation. Our attempts to develop a swine orthotopic ITx model encountered severe surgical and postoperative complications that required three years of troubleshooting with various modifications. The lessons learned now enable us to achieve routine long-term post-ITx survival, with which we have been able to carry out experiments achieving transplant tolerance with mechanistic analysis (results to be reported elsewhere). Mastering this model not only provides a means of gaining knowledge for advancing the field of transplantation but also holds the potential to improve the clinical care of intestinal transplant patients.

## Methods

### Donor and recipient selection

**Figure 1:**
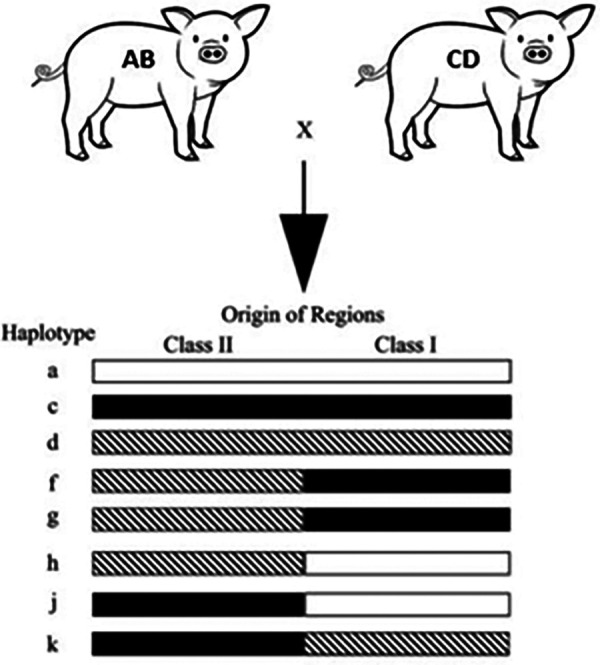
Miniature swine genotype.

We utilized miniature swine. Because of the advantages discussed above, the use of miniature swine provides a unique opportunity to closely mimic the human transplant scenario, allowing researchers to gain essential insights into surgical techniques, immunosuppressive strategies, and post-transplant complications before these procedures are translated to clinical settings. Additionally, our herd of Sachs miniature swine has been inbred to generate lines with three fixed major histocompatibility complex (MHC) class I and class II genotypes (Figure 1). This allows researchers to transplant across defined genetic mismatches to study immunological responses in one- or two-haplotype mismatches of either class I or class I as well as class I-only mismatches, class II-only mismatches, and full MHC mismatches, which mimic most of the scenarios that human recipients could face.

Our pair selection is based on degree of histocompatibility, which allows us to study the mechanism in tolerance and rejection in intestinal transplantation. Blood type should be matched.

### Immunosuppression regimen

**Figure 2:**
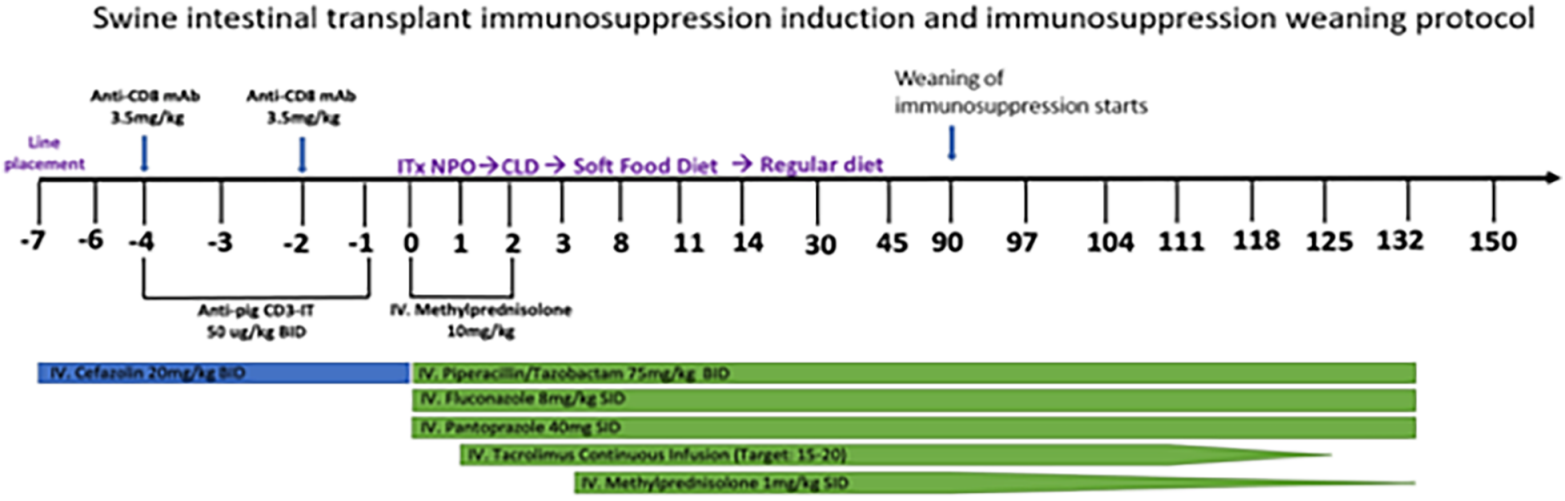
Immunosuppression protocol.

To make the experiment clinically relevant, we designed an immunosuppression regimen (Figure 2) that mirrored the clinical regimen used for human ITx recipients at our institution. One modification was necessary in designing this protocol: T cell depletion was performed with a preoperative regimen of Anti-CD3-Immunotoxin 50 µg/kg IV twice a day and anti-CD8 mAB (3.5 mg/kg IV and preoperative days −2 and −4), since T cell depletion with thymoglobulin was only 25% as effective in swine than in humans (data not shown). Anti-CD3-Immunotoxin is a recombinant immunotoxin targeting porcine CD3, engineered by utilizing a diphtheria toxin foundation (9, 10). This construct combined the truncated diphtheria toxin DT390 with two tandem identical single-chain variable fragments (scFv) from the anti-porcine CD3 monoclonal antibody 898H2-6-15. When combined with the anti-CD8 mAb, this conjugate induces transient yet significant depletion of circulating T cells In swine transplantation models (9, 10).

### Surgical technique

Both the donor and recipient miniature swine undergo general endotracheal anesthesia using 10 mg/kg ketamine, 50 mcg/kg/min propofol infusion, and continuous inhaled 1%–2% isoflurane in 100% O_2_, to ensure they are unconscious and pain-free during the procedure. The donor is euthanized with 100 mg/kg Euthasol (Virbac, Westlake, TX) at the conclusion of organ procurement. The same dose is used to euthanize the recipient when they have reached endpoints later in the course of the experiment.

#### Donor

Donor animals are fasted for 24 h prior to surgery to decrease the stool burden. After anesthesia induction, the abdomen and neck are prepped and draped. An indwelling central venous catheter is first inserted through a paramedian neck incision carried down through the subcutaneous layers to identify the external jugular vein lateral to the sternocleidomastoid muscle. The vessel is incised and cannulated with IV tubing, which is then secured in place. The proximal end is used by the anesthesia team.

The abdomen is then opened with a midline incision, making sure in males to avoid the penis, which runs subcutaneously from the prepuce to the scrotum. The peritoneum is carefully entered sharply to avoid any bowel injury. A Balfour retractor is placed for exposure. The spleen is removed to facilitate exposure. This is accomplished by lifting porcine spleen, which courses from the right upper quadrant extending across the abdomen to the left, and ligating all vascular structures, including the splenic artery and vein.

The small intestine of the pig lies on the right side, and the spiral colon lies on the left. Mobilization is achieved by sharply incising the white line of Toldt on both sides. After mobilization, the plane is avascular and can be developed by a combination of blunt and sharp dissection. We continue to mobilize the peritoneal attachments on the cranial aspect of the right side. In this way, the small intestine and posterior aspect of the proximal pancreas are separated from the inferior vena cava and renal vein. On the cranial aspect of the left side, we mobilize the colon and then follow the avascular plane anterior to the left kidney and adrenal gland and posterior to the distal pancreas. This plane leads to the superior mesenteric artery (SMA), palpated in the root of the mesentery, and the left side of the aorta. With no other important structures lying in this area, the aorta can be dissected from the surrounding crus and dense ganglia tissue to identify the roots of the SMA and celiac artery. The celiac artery is more distant cranially from the SMA in swine than the celiac artery is from the superior mesenteric artery in humans. We then dissect the SMA sharply from its surrounding tissue using electrocautery from its origin into the root of the mesentery, where it approaches the superior mesenteric vein (SMV). There are very few vessels, and none of significance, in the area, so the SMA can be easily skeletonized. Once the bowel is completely mobilized, we dissect the distal aorta to get proximal and distal control with either umbilical tape or 0 silk ties for future cannulation. We do not mobilize the rectum, which runs from cranial to caudal along the posterior abdominal wall to the left of midline.

The next step is division of the spiral colon and its mesentery from the small intestine. To do so we eviscerate the colon out of the left side of the abdomen and divide its mesentery between 2 and 0 silk ties from the terminal ileum (20 cm from the cecum) caudally to the origin of the rectum cranially. The mesentery is divided close to the bowel. The important tie is on the major artery and vein supplying the colon, which lie on the cranial aspect of the spiral colon mesentery.

The final step is to mobilize the superior mesenteric vein. This is particularly challenging since it runs through the pancreas, unlike posteriorly in humans, and it is completely encircled by both the duodenum and the rectum due to the pig's natural malrotation. We accomplish this by sharply dissecting the peritoneal attachments of the SMV from the pancreas and dividing the few small pancreatic branches between 4 and 0 silk ties. In this way, the cranial aspect of the SMV can be mobilized from the pancreas up to the level of the splenic vein, which is our division site. To free the SMV from the mesentery of the duodenum and rectum, we first identify a division site for the proximal jejunum and make a window in the mesentery. By progressively moving proximally and dividing the mesentery of all proximal small intestine between 2 and 0 silk ties, we are able to unwind the duodenum and rectum from around the SMV until it is mobilized.

At that point, we heparinize the donor with 200 units/kg IV and cannulate the aorta in the standard deceased donor procurement fashion. We draw blood for assays and for recipient transfusion from the aortic cannula until the blood pressure becomes undetectable (despite temporarily decrease bowel perfusion, this cannot be done once perfusion starts). We then clamp the aorta cranial to the SMA, which we previously exposed, incise the suprahepatic IVC, and ice the bowel. After perfusing with 2 L of lactated Ringers solution with added 5 units of heparin per ml, we sharply excise the ring of aorta containing the root of the SMA by dividing the aorta cranial and caudal to this. We divide the SMV at its confluence with the splenic vein and ligate and divide the proximal and distal small bowel at the locations where we previously created mesenteric windows. The graft is then brought to the recipient side.

#### Recipient

The recipient undergoes central venous line placement using a Groshong dual lumen catheter (BD-23572) on day −7, followed by 3 days of intravenous antibiotic prophylaxis with Cephazolin. On day −4 to day −1, the recipient receives anti-porcine CD3 immunotoxin 50 ug/kg twice a day. Additionally, the animal receives anti-CD8 mAb on day −4 and day −2 as a daily dose of 3.5 mg/kg.

The recipient is fasted for 24 h prior to transplantation. The incision and mobilization of the small intestine from the posterior abdominal wall is performed on the right side only. We create windows in the proximal aspect of the small intestinal mesentery 20 cm from the ligament of Treitz and in the distal aspect of the mesentery 30 cm proximal to the cecum. The small intestine is divided between 2 and 0 silk ties both proximally and distally, and the native intestine is removed. The caudal aspects of the IVC and aorta are then dissected until they can be fully occluded with Satinsky clamps. The aorta is surrounded by thick connective tissue and should be skeletonized at the site of anastomosis to facilitate sewing.

The recipient is heparinized at 200 units/kg, and the graft is brought into the field. The proper orientation of the bowel is ensured so that the proximal aspect (the location of the mesenteric vessels) is cranial and that the root of the mesentery is not twisted. The IVC is occluded with a Satinsky clamp and then sharply incised to create an opening of the proper size for the SMV. The SMV is anastomosed to the IVC end-to-side in a parachute technique using running 6-0 Prolene. After completion, we clamp the aorta with a Satinsky clamp and judge whether the arterial fit is better with a Carrell patch of aorta or with the entire ring of aorta (the opposite end of the ring is ligated). In later cases, we almost always chose a Carrell patch to minimize the length of the artery and decrease the risk of kinking. The aorta is then sharply incised to create an opening of the appropriate size, and an end-to-size arterial anastomosis is performed in a parachute technique using running 6-0 Prolene.

**Figure 3:**
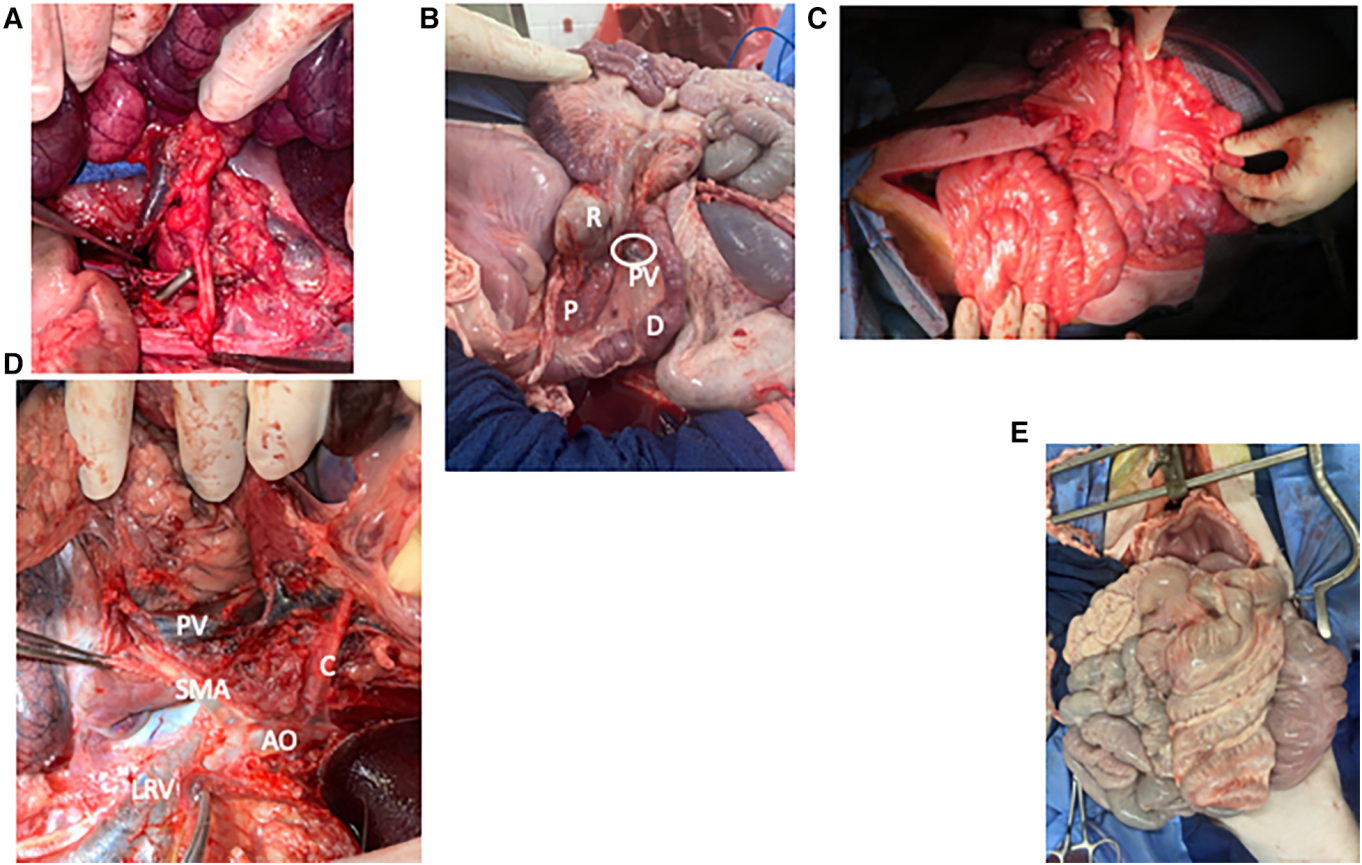
Photo atlas of surgical anatomy. (**A**) Graft being sewn into recipient with graft SMA and its Carrel patch overlying the aorta in the foreground and graft SMV overlying IVC in the background. (**B**) “Corkscrew” of pig foregut showing the duodenum [D] and rectum [R] wrapping around each other, the pancreas [P], and the portal vein [PV and circled]. (**C**) Shortened graft after reperfusion showing comfortable positioning within the recipient's abdomen. (**D**) Vascular anatomy of the foregut from the left side with the spleen, colon, and pancreas reflected. Aorta [AO] gives off the celiac artery [C] and superior mesenteric artery [SMA], which overlies the left renal vein [LRV]. As it enters the mesentery, the SMA lies close to the portal vein [PV]. (**E**) Frontal view of the pig abdominal cavity with the malrotated small intestine entirely on the pig's right and the spiral colon entirely on the left.

The graft is then allowed to reperfuse. There is often a drop in blood pressure at that point as the blood volume fills the large graft. This can be treated with fluid or blood taken from the donor. Pressors may be used if needed, but volume replacement is a more appropriate treatment for fixing the root of the problem as well as avoiding microvascular constriction in the graft. The graft is trimmed proximally and distally to ensure a good fit and presence of only well-perfused graft. The bowel anastomoses are then performed in two layers in the usual hand-sewn fashion. This may be performed end-to-end, end-to-side, or side-to-side according to the judgement of the surgeon (Figure 3).

**Figure 4:**
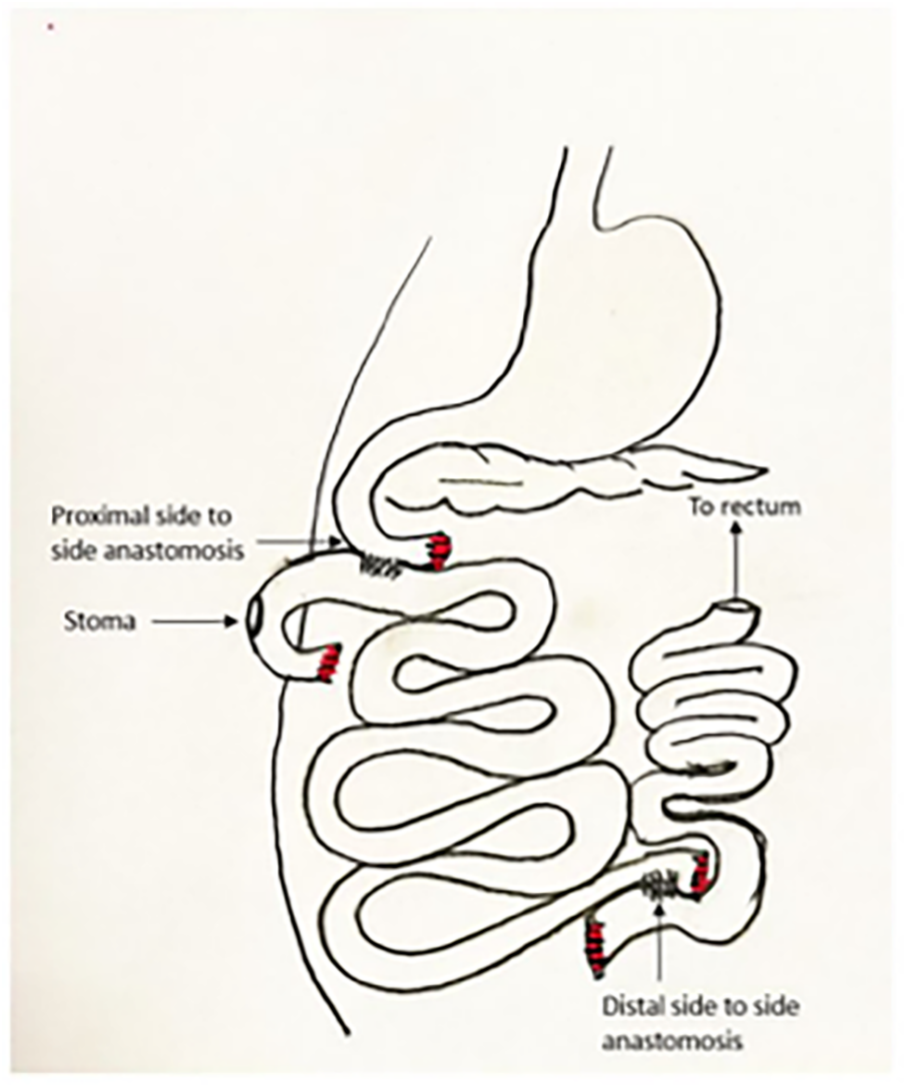
Schema of bowel positioning and anastomoses.

Of note, we use the most proximal aspect of the graft as a defunctionalized chimney for mucosal inspection and endoscopy, so we choose the proximal site at a place where the mesentery permits maximum mobility for a stoma, and the site of proximal anastomosis is 40 cm distal to this. The proximal end is externalized through a stoma site where it can reach without tension, and the spleen is replaced in its normal location. Before closing, the mesenteric defect is closed, the stoma is tacked to the abdominal wall as it passes through the posterior fascia, and the abdomen is generously irrigated with warm saline. The abdomen is then closed in the usual surgical fashion. After closure, the stoma is matured in the fashion of a loop, although the most proximal portion of bowel is sutured closed as a blind end (Figure 4). Endoscopy is performed in a manner similar to an ileoscopy through a stoma in a human subject with the addition of light sedation for the animal's comfort. We use the same gastroscope used in the clinic and advance into the stoma gently using lubrication and minimal amount of insufflation. We take mucosal biopsies using a large biopsy forceps. Hemostasis occurs spontaneously. We remove the scope while suctioning to decrease distension.

### Postoperative management

The recipients are extubated in the operating room and returned to their cage, where they are placed prone on blankets and warmed by a space heater. Postoperative analgesia is provided by long-acting IM buprenorphine. They are monitored hourly for the first 24 h, and point of care venous blood gas results, including electrolytes, hemoglobin, creatinine, and lactate, are checked every four hours overnight and then every 6–8 h during the following day. Intravenous fluid consisting of 5% dextrose with 0.45% normal saline is administrated at ½ maintenance rate for the first 24 h and then discontinued on the evening of postoperative day 1. The recipients are given a liquid diet on postoperative day 2 and solid food on postoperative day 3. The remainder of management is depicted in Figure 2.

## Results

### Outcomes, survival, and causes of failure

Of the total of 22 recipients, the average survival was 55 days with a median of 12.5 days. The longest survival was 551 days, and the shortest was 0 day (Table 1).

**Table 1:** Survivor days and cause of death.

No.	Days survived	Reason for endpoint
1	8	Pulmonary embolism
2	1	Venous thrombosis
3	13	Pulmonary embolism
4	63	Weight loss >30%
5	551	Study ended
6	28	Bowel obstruction
7	20	Hyperthrophic cardiomyopathy
8	13	Pulmonary embolism
9	7	Volvulus around distal stoma
10	2	Venous kinking
11	0	Hemorrhage from SMV tear
12	12	Infection
13	3	Pulmonary edema
14	23	GVHD
15	56	Broken femur
16	9	Acute cellular rejection
17	174	Severe weight loss
18	11	Infection
19	13	Acute cellular rejection
20	176	Acute cellular rejection
21	12	Acute cellular rejection
22	10	Acute cellular rejection

Surgical complications were the cause of death for five recipients. Seven others died from medical complications. Ten recipients managed to survive the perioperative period to reach either immunological or experimental endpoints (Table 2). By analyzing our complications, we learned several important lessons that eventually improved postoperative survival from less than 2 weeks to routine long-term survival. The modifications that were necessitated by complications and that facilitated long-term survival are enumerated below.

**Table 2:** Indication for euthanasia in 22 recipients.

Indication for euthanasia	Surgical technique error (number of recipients)	Medical complication (number of recipients)	End point (number of recipients)
Number of recipients (*n* = 22)	5	7	10

### Modifications in response to surgical complications

In this study, four pigs required euthanasia due to various surgical complications. These included hemorrhagic shock, intra-operative thrombosis, bowel obstruction, and kinking of blood vessels resulting in bowel necrosis. In response to these incidents, and based on other lessons learned during each iteration, we incorporated the following specific modifications in our surgical protocols to address these challenges.

#### Proximal stoma placement

One subject died from a bowel obstruction on postoperative day 7. In this pig, unlike all others, we performed a distal stoma (Bishop-Koop type in the distal ileum) in response to a prior case in which there was high output from a proximal stoma, which caused losses that resulted in malnutrition. We found that the small intestine volvulized around this stoma, and another research group has experienced similar outcomes with distal stoma creation (L. Gonzalez, personal communication). In future cases we reverted back to proximal chimney stomas outside the enteric stream. These stomas are caudal to most of the small intestine and have never caused obstructions in our experience.

#### Loop stoma

We originally constructed the stoma in the form of an end ostomy. The stoma opening closed quickly in this form due to the thick surrounding pig skin. Subsequently we switched to a loop format, with the proximal end sutured closed and folded to create a loop with a blind end on the proximal side (Figure 4). We had no further issues with the stoma with this modification.

#### Vascular modifications

Three subjects died of intraoperative vascular complications. One experienced massive hemorrhage from tearing of the donor SMV during venous anastomosis. A second experienced venous thrombosis. A third had prolonged ischemic time caused by kinking of the vein, which caused venous thrombi and the need to revise the anastomosis several times. All of these complications could be attributed to long length of the donor vein, which led to kinking, twisted anastomosis, and easy tearing due to the weight of the intestine suspended from the long and thin vein. Separately, we had three cases in which the dissection of the complicated porcine foregut led to inadvertent transection of the SMA. This did not cause death in any animal, but it did require expeditious procurement and reconstruction on the backtable. In response, we adopted the following modifications:

1) Decreased length of donor vessels: We decreased length of the donor vein from 4 to 6 cm to about 3 cm. This allowed us to prevent kinking and tearing and also helped to keep the orientation correct. To match with the shorter vein, we replaced use of the ring of donor aorta as inflow with a Carrell patch containing the origin of the SMA (Figure 3A).
2) Shortening of foregut: Choosing a more distal location of the foregut to make the window for our proximal division provided three benefits. First, it was significantly easier to ensure that the mesentery was straight and that the graft was in the proper conformation. Second, it allowed greater mobilization of the SMA and SMV from the bowel, since the proximal bowel wraps around these vessels (Figure 3B). This made the anastomosis easier and decreased the risk of inadvertent vascular injury or kinking. Finally, it decreased the overall mass of the graft, which was helpful for causing less stretch on the graft vein and less risk of compartment syndrome postoperatively.
3) Decrease length of graft small intestine by approximately 1/3: Several recipients died from pulmonary complications. While there were likely medical factors related to these events, which we discuss below, we also noticed that these cases involved some of the highest donor-to-recipient size disparities, meaning that the recipient received a higher volume of graft relative to their size. We were concerned that there might have been a component of abdominal hypertension, especially as the graft bowel became distended and edematous in the early transplant period. These pulmonary issues resolved with both our medical modifications as well as shortening of the graft bowel. We therefore recommend using grafts from smaller donors and shortening the graft length by approximately 1/3. In pigs, this brings the total length from 300–400 cm down to approximately 200–270 cm (Figure 3C). Colon is not reduced since it is not from the donor.
4) Do not ligate or divide recipient SMA or main branches: The recipient surgeon ligated and divided the SMA in one case, which led to ischemia of the distal foregut and much of the colon, requiring a partial duodenectomy and a colectomy. Unlike humans, the SMA supplies a significant portion of the pig foregut and hindgut and should be preserved.
5) Use caution when dissecting distal SMV: The SMA rises from the aorta to the root of the mesentery at the distal SMA, just below the pancreas, where it then runs to the left and deep to the SMV. It is very easy to injure in this area, especially since it lies behind the structure that is being dissected and is surrounded by ganglia tissue that can resemble arterial vessels (Figure 3D).

#### Other surgical modifications

We made several other modifications that improved our approach:

1) Donor: Bowel position during SMV dissection: Because of the swine foregut malrotation (Figures 3B,E), dissecting the SMV from proximal to distal is facilitated by rotating the small intestine from the right to the left, which flattens the course of the SMV and exposes the vessel. However, the amount of rotation required when dissecting the most distal portion of the SMV kinks the more proximal aspect of the SMV, causing impaired venous return. In some cases, once the small bowel is completely mobilized, it is easy to rotate the bowel 360 degrees without realizing it. Therefore, it is important to watch the color of the small intestine during this step and to restore the small intestine to the right side if outflow is compromised. It is also important to make sure the bowel is in its natural position prior to perfusion to ensure that the organs receive an adequate flush.
2) Donor: Division of colon mesentery: There is a short distance from the origin of the colon's major vascular pedicle to the colon wall. Therefore, it is important to divide the colon mesentery close to the colon itself to avoid compromising the CMA and SMV.
3) Recipient: Forego gastrostomy tube: We learned that gastrostomy tube insertion not only increases the operating time and delays recovery, but also that these tubes all became clogged or dislodged within the first two weeks.
4) Recipient: Leave rectum undissected: We learned that mobilizing the rectum from the posterior abdominal wall was a counterproductive step. Not only was it not necessary, but leaving the rectum *in situ* prevented it from becoming entangled with the small intestine.
5) Maintain consistent team: Because of the lessons learned and the muscle memory built with each case, our process improved. Surgery was quicker and more efficient, and anesthesia recovery times were faster.

### Modifications in response to medical complications

Upon analyzing post-transplant complications, a subset of recipients (*n* = 10) encountered medical complications that led to euthanasia. Six out of these ten recipients survived less than 30 days post-transplantation. Five of these died from cardiopulmonary events, with a combination of pulmonary edema and pulmonary embolism in four and hypertrophic cardiomyopathy in a fifth. Two others died of sepsis.

#### Cardiopulmonary modifications

Our modifications primarily concerned the amount or composition of intravenous fluid given to the recipients.

1. Minimize intravenous fluid: One of the most effective interventions was minimization of intravenous fluid during the surgery and postoperatively. Mimicking human protocols, we initially used fluid at maintenance rates and had a low threshold to bolus, especially for elevated lactate and other signs of hypoperfusion. However, we experienced several episodes of fatal pulmonary edema/effusion and congestive heart failure until we tried restricting fluid to no more than half maintenance rates and often less. While we had no further cardiopulmonary complications after instituting this modification, we also had no kidney injury or ischemia from hypoperfusion with the decreased rates.
2. Change perfusion fluid: Our original perfusion fluid was University of Wisconsin (UW) solution (ViaSpan). After experiencing complications with post-transplantation cardiomyopathy and thromboses, and that swine are more hypercoagulable compared with humans in our experience and in the literature (11), we switched to using lactated Ringers with 5 units of heparin per ml added. We also added 2 units/ml of heparin to our continuous tacrolimus infusion. We had no further cardiopulmonary events after making this change.
3. Change in anti-CD8 dosing: One recipient displayed acute pulmonary edema immediately following anti-CD8 monoclonal antibody (mAb) administration, leading to consideration a possible cytokine release syndrome caused by the antibody. To mitigate this, we changed the second dose of anti-CD8 mAb to before the transplant, when pigs would be less susceptible to complications from inflammation or changes in fluid balance.

#### Infection control modifications

Due to septic complications in two recipients, we extended our antibiotic course until after indwelling lines were removed, and we transitioned our catheter selection from the Hickman dual lumen central catheter to the Groshong dual lumen central catheter. The Groshong catheter is equipped with robust tubing, particularly suited for porcine subjects. Furthermore, the presence of a unidirectional valve in the Groshong catheter design effectively prevents complications, including air embolism and retrograde blood flow, which can lead to the formation of blood clots. The mitigation of these issues significantly reduces the likelihood of subsequent interventions, such as line replacements, thereby enhancing the overall efficacy and safety of the experimental procedure.

## Discussion

In conclusion, we have established a swine model of orthotopic intestinal transplantation with reliable long-term survival. Some of the lessons learned, for example regarding fluid balance and type of perfusion solution, are also likely applicable to other large animal transplantation models.

This model has permitted us to carry out novel immunological experiments that would not otherwise be possible. While our scientific results will be reported in greater detail elsewhere, benefits have so far included the identification of two separate regimens that produced tolerance after intestinal transplantation.

Large animal transplant research is expensive, resource-intensive, and technically difficult. Post-transplantation management, which is complex at baseline, is even more difficult in a veterinary setting with non-verbal patients, different physiology, and fewer diagnostic and therapeutic resources. Therefore, the margin of acceptable error is small. We hope that our experiences will be helpful for other research groups.

## Data Availability

The raw data supporting the conclusions of this article will be made available by the authors, without undue reservation.
